# Relationship Between Temporal Variability in Pupils and Neurological Prognosis in Out-of-Hospital Cardiac Arrest Patients

**DOI:** 10.7759/cureus.104975

**Published:** 2026-03-10

**Authors:** Ryo Ichibayashi, Yoshimi Nakamichi

**Affiliations:** 1 Department of Emergency and Critical Care Medicine, Toho University Omori Medical Center, Tokyo, JPN; 2 Division of Emergency Medicine, Department of Internal Medicine, Toho University Sakura Medical Center, Chiba, JPN

**Keywords:** neurological prognosis, neurological pupil index, post-cardiac arrest, quantitative pupillometry, temporal variability

## Abstract

Background

Quantitative pupillometry has been widely used for neurological prognostication after cardiac arrest. However, the timing and frequency of pupillary assessment vary considerably across studies, and the clinical significance of temporal fluctuations in pupillary parameters under intensive care conditions remains unclear. This study aimed to evaluate temporal variability in serial quantitative pupillometry during the early post-cardiac arrest period and to explore its relationship with neurological prognostication.

Methods

This retrospective observational study included adult patients with out-of-hospital cardiac arrest who achieved return of spontaneous circulation (ROSC) and underwent quantitative pupillometry for at least 72 hours at Toho University Omori Medical Center, Tokyo, between October 2018 and March 2020. Pupillary measurements were initiated within three hours after resuscitation and performed every six hours using an automated pupillometer. The Neurological Pupil Index (NPi) and constriction rate (CH) were analyzed bilaterally. Temporal variability was defined using clinically established thresholds for abnormal changes. Neurological outcomes at 90 days were classified according to the Cerebral Performance Category (CPC), with CPC 1-2 considered good and CPC 3-5 poor.

Results

A total of 27 patients were included in the final analysis; 11 (40.7%) had good neurological outcomes and 16 (59.3%) had poor outcomes. Temporal variability in NPi and CH was observed throughout the 72-hour observation period in all patients, regardless of neurological outcome. Although right NPi at 72 hours showed a statistically significant difference between outcome groups, no consistent or reproducible differences in NPi or CH were observed across time points, between both eyes, or across pupillometric parameters. Persistently absent pupillary responses (NPi = 0) were associated with poor outcomes; however, isolated or fluctuating pupillometric values did not reliably predict neurological prognosis.

Conclusions

Quantitative pupillometric parameters exhibit substantial temporal variability in patients after cardiac arrest under intensive care conditions. Fixed time-point assessment of pupillometry may therefore be insufficient for reliable neurological prognostication. Serial and dynamic evaluation, particularly the identification of persistently absent pupillary responses, may provide more clinically meaningful information than isolated measurements. These findings should be considered hypothesis-generating.

## Introduction

Quantitative pupillometry is a non-invasive method that offers greater accuracy, reproducibility, and reliability than manual methods [1,2]. In addition to measuring pupil size and light reflex, it can evaluate constriction rate (CH), reaction time, mean constriction velocity, maximum constriction velocity, and mean dilation velocity. The Neurological Pupil Index (NPi), derived from seven parameters, is a numerical score that indicates the severity of neurological conditions. A value below 3, along with a difference of at least 0.7 between the left and right eyes, is considered indicative of poor neurological prognosis [3,4].

Evaluation by quantitative pupillometry has been reported for various purposes, including detecting the depth of analgesia and sedation levels [5,6], assessing the relationship with intracranial pressure and cerebral edema [7], evaluating the prognosis of patients undergoing brain hypothermia [8], predicting the clinical prognosis of patients with acute lesions [9], and assessing the neurological prognosis of post-cardiac arrest (post-CA) patients [3,8,10]. It has also been reported that the neurological prognosis of patients after cardiac arrest is reflected by the NPi and CH values, which are measured two to six times within 72 hours after return of spontaneous circulation (ROSC) [3,9].

On the other hand, temporal variability in pupil size has been reported [11]. However, investigating temporal variability in healthy individuals is challenging, and evidence is insufficient. If temporal variability is observed, quantitative pupillometry at regular intervals may be inadequate to predict patients' neurological prognosis after cardiac arrest. Still, recording pupils regularly for 24 hours is possible in sedated patients. This study aimed to evaluate temporal variability in serial quantitative pupillometry during the early post-cardiac arrest period and to explore whether such variability may complicate neurological prognostication based on fixed time-point measurements.

## Materials and methods

Patients and methods

Study Design and Setting

A retrospective observational study was conducted on 27 patients with cardiac arrest who were transported to the emergency center at Toho University Omori Medical Center between October 2018 and March 2020. Our hospital is a tertiary medical facility in Tokyo. The study protocol was approved by the Ethics Committee of the Faculty of Medicine, Toho University (approval number: A24079).

Selection of Participants

The study included 82 patients with out-of-hospital cardiac arrest who were transported to the emergency center and successfully resuscitated. Exclusion criteria included patients under 18 years of age, those with a do-not-attempt-resuscitation (DNAR) order at admission, and patients with a Glasgow Coma Scale (GCS) score of 8 or higher at admission [12], as previous studies on neurological prognostication after cardiac arrest have focused on comatose patients with severe impairment of consciousness [3]. Because quantitative pupillometry required 72 hours for assessment, patients who died or were discharged within 72 hours were also excluded.

**Figure 1:**
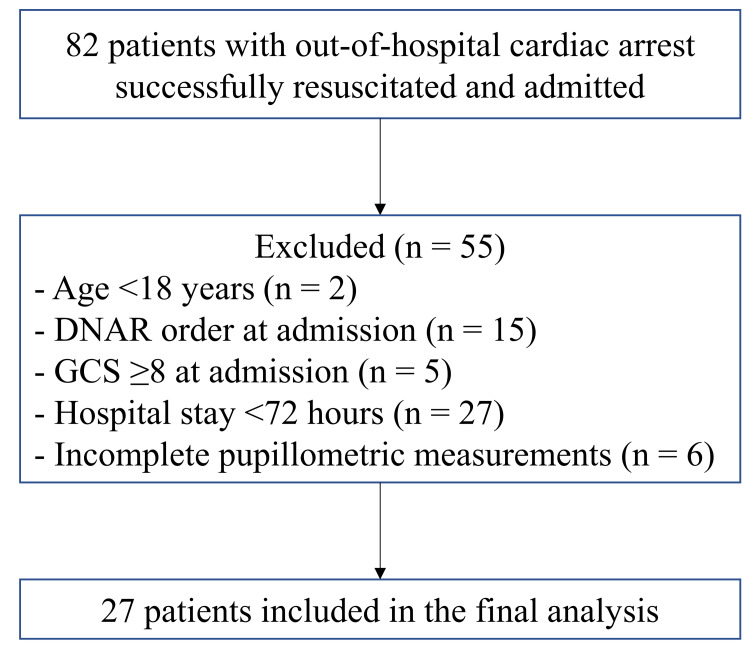
Flowchart of patient selection. Flowchart showing patient selection for the present study. Of 82 patients with out-of-hospital cardiac arrest who achieved return of spontaneous circulation (ROSC), 55 were excluded based on predefined criteria, including Glasgow Coma Scale (GCS) score ≥8 at admission, age <18 years, do-not-attempt-resuscitation (DNAR) order at admission, hospital stay <72 hours, and incomplete pupillometric measurements. Ultimately, 27 patients were included in the final analysis.

Among the excluded patients, five (6.1%) had a GCS score ≥ 8, two (2.4%) were under 18 years of age, and 15 (18.3%) had a DNAR order. In addition, 27 (32.9%) had hospital stays of <72 hours, and six (7.3%) had incomplete measurements. Ultimately, 27 patients were included in the final analysis. The patient selection process is summarized in Figure 1.

Measurement and Data Collection

Quantitative pupillometry was conducted using an automated pupillometer (NPi-200, NeuroOptics Inc., Irvine, CA, USA). It measures the pupil's light reflex by quantifying pupil diameter. From these measurements, the maximum pupil diameter (SIZE), minimum pupil diameter (MIN), CH, reaction time (LAT), mean constriction velocity (CV), maximum constriction velocity (MCV), and mean dilation velocity (DV) were recorded. The difference between maximum and minimum diameter is called the rate of change of the pupillary light reflex, and the NPi is derived from these parameters. In this study, measurements were obtained within 3 hours after cardiac rhythm was restored or cardiopulmonary bypass was initiated. The left and right pupils were measured using the NPi-200 four times daily at six-hour intervals, up to 72 hours after the initial pupillary recording. Previous studies have shown that the NPi and CH can predict neurological prognosis after cardiac arrest [3,4,8,10,13,14]. Therefore, NPi and CH were analyzed for both pupils at each time point. In addition, temporal variability was evaluated for each eye. These records were extracted from the medical records.

Outcome measures

Temporal Variability

During the 72-hour observation period, the highest and lowest values of quantitative pupillometry for each eye were recorded every 24 hours. Temporal variability was defined as having one or more of the following within every 24 hours: a difference in NPi of 0.7 or more, a pupil diameter difference of 1.0 mm or more, or a change in CH of 10% or more. These thresholds were determined based on established clinical criteria: a difference in NPi ≥0.7 is considered abnormal, a pupil diameter difference ≥1.0 mm is considered anisocoria, and a variation in CH ≥10% is considered abnormal. Accordingly, the thresholds used to define temporal variability in this study were set in reference to these existing abnormality criteria [11,15].

Neurological Outcome

Outcome variables included survival rate and neurological outcome at 90 days after resuscitation. Neurological outcome was classified using the Cerebral Performance Category (CPC) scale as follows [3,4]: CPC 1, no neurological abnormality; CPC 2, moderate impairment; CPC 3, severe disability; CPC 4, coma or persistent vegetative state; CPC 5, brain death. CPC 1 and 2 were considered good neurological outcomes, while CPC 3, 4, or 5 were considered poor outcomes. To examine whether each quantitative pupillometry parameter differed between CPC 1-2 and CPC 3-5, there were 11 patients in the CPC 1-2 group and 16 in the CPC 3-5 group.

Statistical analysis

All continuous variables showed a non-normal distribution. Continuous variables were expressed as median (IQR: interquartile range), and nominal and ordinal variables were expressed as percentages. For continuous and ordinal variables, the Mann-Whitney U test was used. The chi-square test or Fisher’s exact test was used for nominal variables. For all tests, a p-value <0.05 was considered statistically significant. GraphPad Prism version 9 for Windows (GraphPad Software, San Diego, CA, USA) was used for the analyses presented above.

## Results

Patient characteristics

There were 11 (40.7%) patients with CPC 1-2 and 16 (59.3%) with CPC 3-5. The patient characteristics are shown in Table 1. In post-cardiac arrest (CA) patients, the median estimated cardiac arrest time (ECAT) was 15 minutes in the CPC 1-2 group and 35 minutes in the CPC 3-5 group, indicating a shorter ECAT in the CPC 1-2 group (p = 0.0068). The number of cases requiring venovenous extracorporeal membrane oxygenation (VA-ECMO) was higher in the CPC 3-5 group. In both groups, cardiac arrest due to cardiac disease accounted for the majority of cases. Target temperature management (TTM) was performed in all cases. In most cases, TTM was performed at 34°C. Sedatives were used in all cases, with midazolam or propofol being administered. Catecholamine was used in all cases, and fentanyl was used as an analgesic (Table 1).

**Table 1:** Patient characteristics Data are presented as count (percentage) or median (IQR： interquartile range) ECAT: estimated cardiac arrest time, VA-ECMO: veno-arterial extracorporeal membrane oxygenation, IABP: intra-aortic balloon pumping, TTM: targeted temperature management, CA: cardiac arrest, GCS: Glasgow Coma Scale, ROSC: return of spontaneous circulation, ED: emergency department, NS: not statistically significant

Variable	CPC 1-2（n = 11）	CPC 3-5（n = 16）	p-value
Age, years（IQR）	64（57-64）	63（53-71）	0.7994
Male gender, n（%）	9（75）	15（94）	0.3324
ECAT, minutes（IQR）	15（8-34）	35（30-46）	0.0068
Shockable rhythm, n（%）	4（36）	9（56）	0.3096
VA-ECMO n（%）	2（18）	10（63）	0.0228
IABP n（%）	4（36）	9（56）	0.3096
TTM at 34 ℃, n (%)	10（91）	14（88）	0.7818
TTM at 36 ℃, n (%)	1（9）	2（12）	0.7818
Etiology of CA	-	-	-
Presumed cardiac cause, n (%)	8（73）	11（69）	0.8240
Non-cardiac cause, n (%)	3（27）	5（31）	0.8240
Sedation	-	-	-
Midazolam, n (%)	5（45）	9（56）	0.8699
Propofol, n (%)	6（55）	8（50）	0.6211
Analgesia	-	-	-
Fentanyl, n (%)	11（100）	16（100）	NS
Catecholamine, n (%)	11（100）	16（100）	NS
GCS after ROSC in ER; median	3	3	NS

Temporal variability of quantitative pupillometric parameters obtained from patients after cardiac arrest

The NPi and CH met the predefined criteria for temporal variability in all patients during the 72-hour observation period, based on threshold-defined fluctuations (difference in NPi ≥0.7, pupil diameter difference ≥1.0 mm, or change in constriction rate ≥10%).

**Figure 2:**
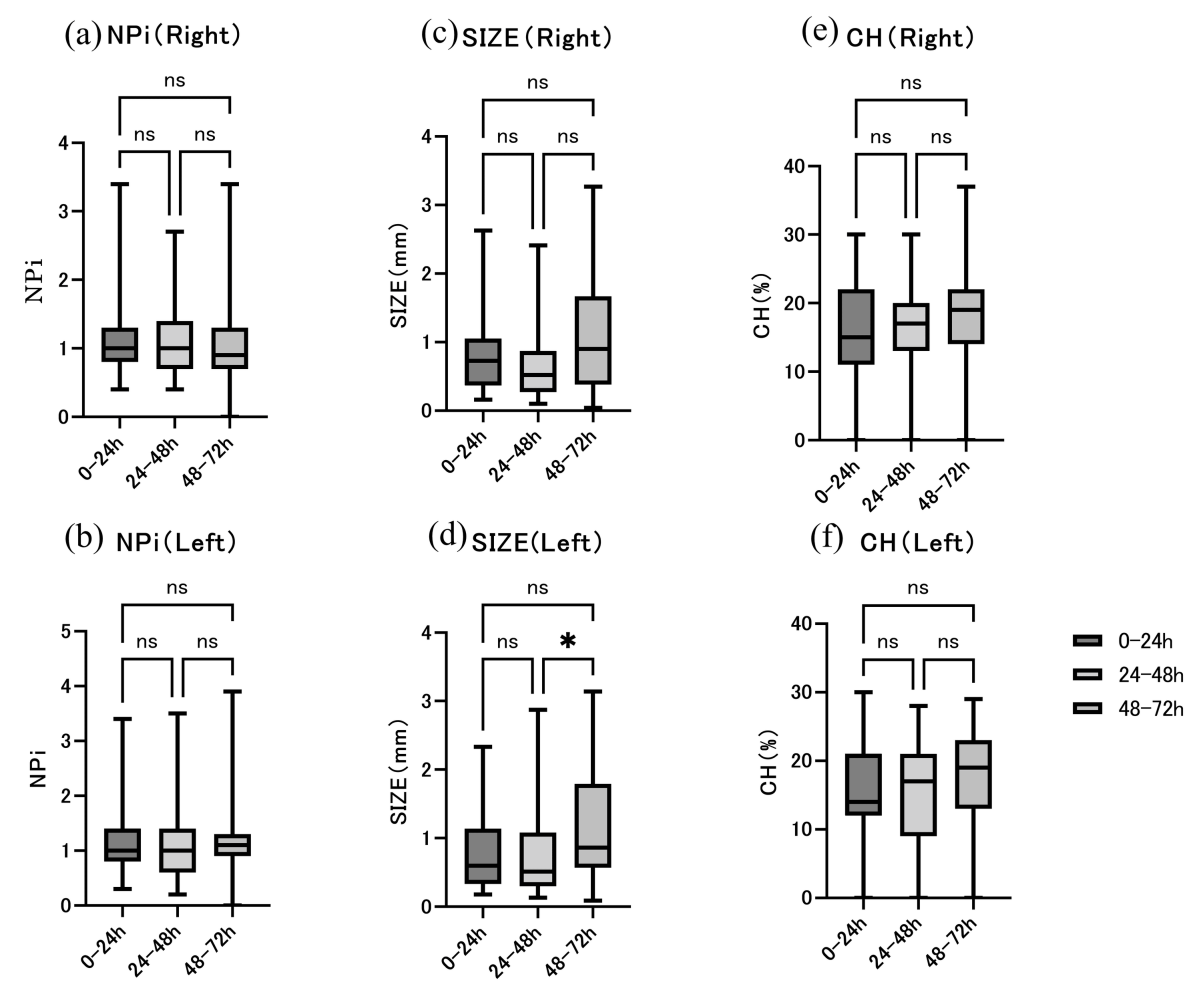
Temporal variability of quantitative pupillometry parameters during the first 72 hours after return of spontaneous circulation (ROSC). (a) Right Neurological Pupil Index (NPi), (b) left NPi, (c) right pupil diameter (size), (d) left size, (e) right constriction rate (CH), and (f) left CH measured during 0–24, 24–48, and 48–72 hours after resuscitation. Data are presented as median values with interquartile ranges. Statistical comparisons were performed between time intervals. “ns” indicates not statistically significant (p ≥ 0.05). An asterisk (*) indicates statistical significance (p < 0.05). NPi: Neurological Pupil Index, CH: constriction rate

By contrast, the pupil diameter (size) did not consistently meet the predefined variability criteria, although a statistically significant change in median fluctuation was observed in the left pupil between 24-48 and 48-72 hours (Figure 2). It should be noted that temporal variability in this study was defined by threshold-based changes within each patient, rather than by statistical differences between time points.

Relationship between the NPi and CH measured by quantitative pupillometry and neurological outcome

The NPi and CH were compared at three, 24, 48, and 72 hours after ROSC between the CPC 1-2 and CPC 3-5 groups (Table 2). Within three hours after ROSC, there were no significant differences in right NPi (p = 0.3325) or left NPi (p = 0.2987). Similarly, no significant differences were observed at 24 hours (right p = 0.8935, left p = 0.3343) or at 48 hours (right p = 0.6166, left p = 0.6162). At 72 hours, the right NPi was significantly higher in the CPC 1-2 group compared with the CPC 3-5 group (median 4.40 vs. 3.90, p = 0.0425), whereas the left NPi did not reach statistical significance (median 4.30 vs. 3.75, p = 0.0903). For CH, no statistically significant differences were observed between the groups at any time point, including 72 hours (right p = 0.0648; left p = 0.1438).

**Table 2:** Comparison of the Neurological Pupil Index (NPi) and constriction rate (CH) between CPC 1–2 and CPC 3–5 groups at three, 24, 48, and 72 hours after return of spontaneous circulation (ROSC). Data are presented as median (interquartile range). Statistical comparisons were performed between the CPC 1–2 (good neurological outcome) and CPC 3–5 (poor neurological outcome) groups using the Mann–Whitney U test. One patient in the CPC 3–5 group exhibited persistently absent pupillary response (NPi = 0) from 54 hours after ROSC onward, and CH was not measurable. A secondary analysis excluding this case did not change the statistical significance of the results. NPi: Neurological Pupil Index, CH: constriction rate, CPC: Cerebral Performance Category, ROSC: return of spontaneous circulation

Variable	CPC 1-2（n = 11）	CPC 3-5（n = 16）	p-value
NPi within three hours after resuscitation (right)	4.40（3.90-4.60）	4.15（3.70-4.40）	0.3325
NPi within three hours after resuscitation (left)	4.20（3.40-4.45）	4.00（3.48-4.30）	0.2987
NPi after 24 hours (right)	4.30	4.20	0.8935
NPi after 24 hours (left)	4.30	4.15	0.3343
NPi after 48 hours (right)	4.50	4.55	0.6166
NPi after 48 hours (left)	4.50	4.30	0.6162
NPi after 72 hours (right)	4.40	3.90	0.0425
NPi after 72 hours (left)	4.30	3.75	0.0903
CH within three hours after resuscitation (right)	12.0	10.5	0.8553
CH within three hours after resuscitation (left)	12.0	10.0	0.7248
CH after 24 hours (right)	12.0	13.0	0.7061
CH after 24 hours (left)	15.0	13.0	0.4726
CH after 48 hours (right)	17.0	16.0	0.7066
CH after 48 hours (left)	17.0	15.5	0.4417
CH after 72 hours (right)	28.0	13.0	0.0648
CH after 72 hours (left)	17.0	10.5	0.1438

In one patient in the CPC 3-5 group (n = 16), the NPi decreased to 0.5 at 54 hours after ROSC and subsequently remained at 0; CH was not measurable thereafter. Excluding this case did not alter the statistical results.

 Cases with an NPi ≤ 2 were compared between the CPC 1-2 and CPC 3-5 groups, as shown in Table 3. Fisher's exact test showed no significant difference between the two groups (p = 0.0536).

**Table 3:** Neurological prognosis in patients with NPi ≤ 2. Fisher's exact test showed that in the CPC 1–2 groups, NPi never fell below 2 during the study period. On the other hand, in the CPC 3–5 group, 37.5% of patients had NPi ≤ 2. Fisher's exact test, p = 0.0536 NPi: Neurological Pupil Index, CPC: Cerebral Performance Category

Data analyzed	CPC 1-2 (n = 11)	CPC 3-5 (n = 16)
NPi ≤ 2	0（0%）	6（37.5%）
NPi > 2	11（100%）	10（62.5％）

## Discussion

Many studies have been published to predict the neurological prognosis of patients after cardiac arrest who have been successfully resuscitated. Some of these studies have reported that quantitative pupillometry helps predict prognosis [3,4,8,10,13,14]. In these studies, the NPi and CH were measured early after cardiac arrest to aid in predicting prognosis. However, the timing of the first measurement varies: on the first day after ROSC, immediately after ROSC, or within six hours of ROSC. The measurement interval is also not standardized, ranging from every six hours to every 24 hours, or even once. However, it has been consistently reported that early measurements of the NPi and CH using quantitative pupillometry affect the prediction of neurological prognosis. Such heterogeneity in the timing and frequency of assessment suggests that temporal fluctuations in pupillary parameters may influence the interpretation of quantitative pupillometry.

In addition, previous reports have indicated that an NPi of 2 or less, or particularly 0, is associated with a poor neurological prognosis [3,4,16]. However, cases with poor outcomes have also been observed even when NPi exceeds 2. Therefore, neurological prognostication is typically conducted using a multimodal approach that includes electroencephalography (EEG), neuron-specific enolase (NSE), and somatosensory evoked potentials (SSEP). Despite this, accurately predicting neurological outcomes remains challenging [3,16]. In summary, quantitative pupillometry alone is insufficient to reliably predict neurological prognosis following cardiac arrest.

Several challenges are associated with predicting neurological prognosis using quantitative pupillometry. First, the pupil should be dilated during cardiac arrest, and NPi should be 0. This is because, in principle, the sympathetic and parasympathetic nerves control pupil constriction and mydriasis, and autonomic nerves and cardiac arrest are considered states without nervous excitation. However, a study by Behrends et al. detected a pupil light reflex during resuscitation in 83% of patients during cardiac arrest. It was concluded that patients whose light reflex lasted more than 5 minutes had an excellent neurological prognosis [17]. If the short period required for resuscitation is related to neurological prognosis, it is debatable when quantitative pupillometry should be performed. Neurological prognosis will be extremely difficult if the evaluation differs by minutes or hours.

In addition, epinephrine, commonly used in resuscitation, and noradrenaline, used to maintain blood pressure after resuscitation, are sympathomimetic amines that dilate the pupils in response to light. A light reflex does not accompany dilation of the pupils due to cardiac arrest. Similar pupil findings are seen in cases of poisoning due to anticholinergic drugs, but if poisoning is ruled out, this indicates that autonomic function has stopped. Therefore, the detection of the light reflex during resuscitation must account for the effects of administered drugs. Thus, careful attention should be paid to pupil evaluation during and after resuscitation when sympathomimetics are used. In addition to sympathomimetics, sedatives, narcotics, and lighting conditions are also said to affect quantitative pupillometry [15,18,19]. Because these drugs are administered for several days after resuscitation, prognosis evaluation immediately after resuscitation must be performed with even greater caution. These external factors may further amplify the intrinsic temporal variability of pupillary responses in critically ill patients.

Strengths and clinical implications of this study

A central finding of this study is that pupillary parameters exhibited temporal variability during the first 72 hours after ROSC, irrespective of neurological outcome. To our knowledge, the existence and clinical implications of temporal variability in quantitative pupillometric parameters after cardiac arrest have not been sufficiently discussed in previous studies. Importantly, temporal variability in this study was defined using clinically established threshold criteria (difference in NPi ≥ 0.7, pupil diameter difference ≥ 1.0 mm, or change in constriction rate ≥ 10%) within individual patients. This definition reflects clinically meaningful intra-individual fluctuations and does not necessarily correspond to statistically significant differences between outcome groups at selected time points.

Accordingly, the presence or absence of statistical significance in intergroup comparisons should not be interpreted as equivalent to the presence or absence of temporal variability. Although NPi and CH did not show consistent statistically significant differences between outcome groups at fixed time points, both parameters met the predefined criteria for temporal variability across the observation period, suggesting instability of isolated measurements rather than prognostic discrimination.

In the present study, right NPi at 72 hours after ROSC reached statistical significance between the CPC 1-2 and CPC 3-5 groups. However, this isolated finding should be interpreted with caution. Statistical significance was observed at only one time point and only in the right eye, without consistent differences across other time points, in the left eye, or in constriction rate. Multiple comparisons were performed across several time intervals and parameters, increasing the possibility of a type I error. Furthermore, the relatively small sample size limits the robustness and generalizability of this finding. Importantly, although right NPi at 72 hours differed statistically between outcome groups, the distributions of values overlapped substantially, and no clear threshold for reliable prognostic discrimination could be identified. Therefore, this finding should be considered exploratory rather than confirmatory.

Taken together, these findings suggest that single fixed time-point quantitative pupillometry may not provide reliable standalone prognostic discrimination after cardiac arrest. Rather, the presence of temporal variability may complicate prognostic interpretation when pupillary measurements are assessed without reference to serial trends.

By contrast, the pupil diameter (size) did not consistently meet the predefined variability criteria, although changes in median fluctuation were observed in specific intervals. This distinction suggests that dynamic functional parameters such as NPi and constriction rate may be more susceptible to short-term physiological influences than static measurements of pupil diameter.

In critically ill populations, pupillary measurements may also be influenced by ambient light, sedatives, opioids, catecholamines, and targeted temperature management [15]. Therefore, the observed fluctuations may reflect not only biological temporal variability but also treatment-related and environmental variability. In severely ill patients, the combined effects of intrinsic variability and external influences further complicate neurological prognostication.

A sustained NPi of 0 strongly predicted poor neurological outcome in the present cohort, consistent with previous reports [14]. One case in this study demonstrated persistent NPi = 0, and the neurological prognosis was poor. This finding suggests that persistently absent pupillary responses may still be clinically meaningful, whereas fluctuating values above this threshold require more cautious interpretation.

Overall, these findings should be regarded as hypothesis-generating. They support the view that temporal variability in pupillometric parameters has not been sufficiently discussed and that such variability may limit the reliability of fixed time-point pupillometric prognostication after cardiac arrest.

Limitations

This study has several limitations. First, the sample size was small, limiting statistical power and potentially increasing the risk of type I or type II errors. Second, survivorship bias may have been introduced because patients who died or were discharged within 72 hours were excluded from the analysis due to the requirement for serial pupillometry measurements. As a result, both the most critically ill patients and some early recoverers may have been systematically excluded, which may influence the observed temporal variability and limit the generalizability of the findings. Third, this was a single-center retrospective observational study, which may restrict generalizability. Fourth, multiple comparisons were performed across several time points and parameters without formal adjustment, and therefore, the statistically significant finding for right NPi at 72 hours should be interpreted cautiously.

In addition, prognostic performance metrics, such as sensitivity, specificity, and the area under the receiver operating characteristic curve, were not evaluated. The effects of sedatives, opioids, catecholamines, targeted temperature management, and ambient light conditions may also have influenced pupillary measurements. Although opioids and catecholamines were administered in all cases, dose-dependent and time-dependent treatment effects were not evaluated in this study.

Therefore, these findings should be interpreted as hypothesis-generating rather than definitive, and larger prospective studies are required to determine whether serial quantitative pupillometry improves neurological prognostication after cardiac arrest.

## Conclusions

Patients resuscitated from cardiac arrest may exhibit temporal variability in pupillary responses regardless of neurological outcome. Although a statistically significant difference in right NPi at 72 hours was observed between outcome groups, this isolated finding should be interpreted cautiously and does not establish reliable prognostic discrimination. Therefore, caution is warranted when interpreting prognostic estimates derived from quantitative pupillometry, as fluctuations due to temporal variability may influence pupillary measurements.

Serial and dynamic pupillary observation using quantitative pupillometry may help identify persistently absent pupillary responses, thereby supporting early clinical assessment of severe neurological injury in patients after cardiac arrest. Overall, these findings should be considered hypothesis-generating rather than definitive.
